# Experience of Facility Based Childbirth in Rural Ethiopia: An Exploratory Study of Women's Perspective

**DOI:** 10.1155/2017/7938371

**Published:** 2017-11-22

**Authors:** Yohannes Mehretie Adinew, Netsanet Abera Assefa

**Affiliations:** College of Health Sciences and Medicine, Wolaita Sodo University, Sodo, Ethiopia

## Abstract

**Background:**

In Ethiopia, majority (62%) of pregnant women attend antenatal care at least once, yet only 26% deliver with skilled birth attendants in the available health units. Thus, this study explored beliefs and behaviors related to labour and skilled attendance among the women, their perspectives on health care providers, and traditional birth attendants.

**Methods:**

Sixteen key informant interviews and eight focus group discussions were conducted among purposively selected women who had previous experience of facility based childbirth but gave birth to their most recent child without skilled attendance in the last 12 months. Thematic content analysis was used to elicit and assess the various perspectives of each group of participants interviewed.

**Findings:**

The study participants described a range of experiences they had during childbirth at health facilities that forced them to choose home delivery in their most recent delivery. Three themes and six subthemes emerging from women's description were abusive and disrespectful treatment, unskilled care, poor client provider interaction, noncontinuous care, lack of privacy, and traditional practices.

**Conclusion:**

The abuse and disrespect from providers are deterring women from seeking skilled attendance at birth. Thus the health care providers need to improve client provider relationships.

## 1. Background

Childbirth is a crucial event in the lives of women and represents a time of intense vulnerability. Complications from pregnancy and childbirth are the leading causes of mortality and morbidity for women of child bearing age in developing nations like Ethiopia [1], where institutional delivery is low [2]. Ethiopia has maternal mortality ratio of 412 and child mortality of 59 [1]. Studies have indicated home delivery as the main reason for maternal mortality and morbidity among rural women in Ethiopia [3, 4]. Thus improved access to emergency obstetric and neonatal care can reduce this tragic loss of life and vitality up to 75% [5, 6].

Despite decades of efforts to encourage facility based births, many women continue to deliver at home. For instance, in Ethiopia, access to health service has been greatly improved and maternal services had been free but facility based childbirth is still 26% while skilled attendance at birth is only 28% [1]. Recent studies also suggested that improving access is not sufficient to increase use [7].

Though current government reports showed an improvement in skilled attendance, from 10% in 2011 to 28% in 2016, [8, 9] some women are giving their back to the service once experiencing it. As a result, improving maternal health remains important challenge to the nation [10, 11].

Birth experience is very individual, unique, and unforgettable [12]. It also has lasting insinuation for women's health and well-being [13]. The birth experience could be positive if a woman is served respectfully and provided support in the form of comfort, reassurance, and praise during labour and delivery [14]. In contrast, it could be negative if a woman experiences disrespectful and inhumane treatment, culturally inappropriate care, and poor quality service [15].

Previous studies have described the sociodemographic factors affecting the use of institutional delivery services but little is explored on how sociocultural and psychological factors determine the use of institutional delivery service. Thus, the aim of this study was to explore why some women who had previous experience of facility based delivery care gave birth at home for their most recent child by in-depth understanding of women's previous facility based delivery experience, perspective towards health facilities and service providers with regard to delivery services.

## 2. Methods

### 2.1. Study Design and Area

An exploratory qualitative study was employed. Focus group discussions and in-depth interviews were held with purposively selected study participants who fulfilled the inclusion criteria. The study was conducted in two purposively selected districts Lemo and Gombora of Hadiya Zone, south Ethiopia. Hadiya zone has 21 health units, 12 in Lemo and 9 in Gombora.

### 2.2. Participants

The study participants were women who gave birth to at least one of their previous children in the health facility during the last five years (2011–2015) but gave birth to their most recent child (in the last twelve months prior to data collection) without skilled attendance. Those women with experience of both facility based and home delivery were purposively identified and enrolled in the FGDs and IDIs with the help of health extension workers in the district. Diverse participants were recruited to get good representation (polygamous and monogamous, old and young, and educated and uneducated). A proportional number of FGDs and IDIs were held in the two selected woredas.

### 2.3. Sampling Technique and Procedure

Two kebeles (the lowest administrative unit in Ethiopia) were selected form each woreda (district). Two FGDs and four IDIs were held in each of the four selected kebeles from the two woredas. Each FGD had 8–10 participants. The number of FGDs and IDIs was determined by saturation of information.

### 2.4. Inclusion Criteria

Women who had previous experience of facility based childbirth in the last five years but gave birth to their most recent child at home in the last twelve months prior to data collection were included.

### 2.5. Data Collection Tools and Procedure

Data were collected through FGDs and key informant interviews from March to June 2016. The discussions were audio-taped using a digital voice recorder. The interviews and FGDs were held at sites chosen by the participants. Midwifes familiar with the community norms were recruited for the FGDs and in-depth interviews to elicit detailed responses as the issue was sensitive. The main topics covered were reasons for not seeking delivery care; the decision making process for accessing skilled delivery care; perceptions of the quality of delivery care at available health units; perspectives on planning for birth and emergencies; and the reasons why some women are turning their back to skilled delivery care. Both the FGD and the in-depth interview were guided by an experienced person fluent in the local language (Hadiya) and English.

### 2.6. Data Analysis

All FGDs and key informant interviews were transcribed and translated verbatim from the local language to English by individuals fluent in both languages. The transcripts were entered into Microsoft Word and thematic content analysis was done. Specifically, the coding process involved identifying major themes in each of the transcripts. Identified themes were compared across the transcripts to determine differences and similarities in the perspectives of the study participants on childbirth and the factors influencing women's decisions to seek skilled delivery care. Three themes and six subthemes were identified (Table 1).

### 2.7. Quality Control

Training was given to the data collectors for one day on the objective of the study, confidentiality of information, participant's right, informed consent, and techniques of interview. Before the actual data collection, pretest was conducted in adjacent kebeles to ensure the validity of the tool. The supervisors and the principal investigator made frequent checks on the data collection process to ensure the completeness and consistency of the gathered information. 

## 3. Findings

### 3.1. Demographic Characteristics of Study Participants

Most (69.2%) of participants were between ages 20 and 29. Majority (57.7%) of the respondents had attended primary school, and 7.6% had none. Among the participants, 53.8% had four or more children (Table 2).

### 3.2. Findings from Focus Group Discussions and In-Depth Interviews

FGDs and IDIs were used to learn from the women about their experiences. The study participants described a range of experiences they had during childbirth at health facilities that forced them to choose home delivery in their most recent delivery. Six subthemes emerged from women's description: abusive and disrespectful treatment, unskilled care, poor client provider interaction, continuity of care, lack of privacy, and traditional practices.

### 3.3. Health Provider's Abusive and Disrespectful Treatment

Previous negative experiences with facility births may deter women from delivering at a facility during a future birth. Some women pointed out that they have been treated neglectfully and arrogantly during their labour by the providers. A woman who had 15-day-old baby from an in-depth interview stated that

*…I was saying sisters don't you have a sister, don't you have a mother? Please save my life. They replied me that we are not God to do so. I said yes, you are whom I have next to God. But they did not care to my cries. Thereafter, I lost hope on them and kept saying oh God help me people have refused to help me in Hadiyigna [the local language in hadiya zone]. One interprets what I am saying to the other one and they both were laughing. I wish none of my future generation be attended there*. (IDI, Lemo)

Women reach health facilities with difficulties especially for delivery. But health care providers were sending labouring women back to the community rather than assisting them. A woman from FGD reported,

*It was at night that we went to the health center and he [provider] told me that my labour was yet to come but I knew I was in labour and also that thing has flashed [amniotic fluid]. He [the provider] instructed us to go to any of the neighboring houses and have some warmth possibly from fire which he believes would intensify the labour. It was in the middle of the night; we went to one nearby house and delivered the baby while I was feeling the fire. Thanks to God and families of that house they attended to me*. (FGD 1, Gombora)

Some of the mothers in focus group discussion and key informant interviews have mentioned health provider's bad approach to labouring mothers. Majority of mothers participated explained the issue using the following expression:

*Some of service providers are hostile to a labouring mother. For instance when I gave birth to my first child in the health center I was ashamed to open my leg in front of male nurses during labour. Then one of the health worker said “had it been for sex you would open shamelessly.” It was my fault, had it been in my mom's home I would enjoy the ceremony and give birth with respect. But, I refused them and went to health center just to end up with such insult. This type of arrogant approaches made me to give birth to my second child at home.* (FGD 2, Gombora)

Another participant said,

*If a husband goes to health center with his labouring wife, they interrogate him saying “what did you bring to your wife, why did you come here without food and clothe to her, why did you make your wife pregnant if you have no sufficient income.”* (FGD 1, Lemo)

Women prefer to deliver at home, in a familiar and convenient setting. Some even choose traditional birth attendants (TBAs) over health care providers emphasizing the close bond that they felt with TBAs, due to their status in the community and the trust they developed over years of experience. This relationship often prompted women to desire home-based births attended by a TBA rather than a facility. One women from the in-depth interview said

*the traditional birth attendants will take care of your feelings; they treat you with sympathy. They are well aware of and concerned about our culture, so they never do something that can disappoint you. But those in health facilities act as if they were from another planet. They enjoy your pain and degrade you from humanity. I don't even understand why they are here if they don't respect and serve the needy*. (IDI, Gombora)

### 3.4. Unskilled Care

Women were not satisfied with the care they received at the health facilities and had great complaint over the providers' skill. One of the in-depth interview participant said

*…the provider was too young and has no confidence on what he was doing; I was scared to death when I saw his shaking hands and sweating face. He was very nervous, he took one instrument then put it down and took another and I was like oh God have mercy on my soul. Another nurse came inside and yelled at him and started arguing on the procedure as I was suffering the pain and blood loss; they call a third female nurse from home and she helped me out. But, days after my discharge I developed infection and was referred to hospital due to the unclean procedure*. (IDI, Lemo)

One woman from the FGD said

*… they cut my genitalia during labour [episiotomy] but stitched it without giving me any anti pain; I tolerated it and went home. After hours the wound started to bleed heavily then I woke up in hospital bed. The doctor told me I survived the impossible*. (FGD, Gombora)

Women would have been confident if they had heard correct findings on their labour status. One woman from FGD was told conflicting reports from the providers.

*I went to the health center during the day and I was told by the providers that I should stay In the facility as my labour was in progress but when the night shifts came they [providers] told me to go back home saying that my labour was false labour. However, I gave birth hours later in my home*. (FGD, Gombora)

### 3.5. Poor Client Provider Interaction

Upon arrival to a facility, women often experienced delays in care provision and health workers were often slow to respond to client needs. Health workers often did not communicate with the woman or her family on the progress of labour. Women were glad to be welcomed to the health facility during labour. Nevertheless, some women who went at night were unlucky to be received. A woman from an in-depth interview said

*By the time we got to the hospital, a janitor said the providers were asleep…they [providers] may or may not wake up or may nag at you so you better ask them very sincerely. And my husband knocked the duty station door, they [providers] did not answer, he knocked it again they did not answer and when he [husband] knocked for the third time he knocked the door very well. Now, they [providers] came out looking sleepy and upset…and said why are you knocking like this? My husband replied, my wife is in labour and she is about to die, save her life please. The provider replied, “do we look like God?”* (IDI, Lemo).

### 3.6. Noncontinuous Care

Lack of periodic assessments during provision of care was the other complaint. Women would have been happier if the providers checked on them intermittently throughout the duration of their stay. However, few women were left unattended for a long time in the labour room. A woman from a FGD stated

*…they [providers] directed me to the bed and then nobody has checked on me. She [provider] came when the baby was about to fall down*. (FGD, Lemo)

### 3.7. Lack of Privacy

Lack of privacy was another concern raised by the participants. Women are not comfortable to expose their private areas to health professionals. Women perceived TBAs as providing high quality delivery care, often emphasizing the supportive and emotional role that TBAs play. One participant said,

*In health center there is no privacy at all. Everybody who comes in and out of the delivery room watches my naked body and inserts their fingers in to my genitalia including the male. Had it been at home let alone inserting finger no one will see my privates as the area will be dark enough to provide me the maximum privacy and only females will attend me….* (FGD, Gombora)

### 3.8. Traditional Practices

In key informant interviews and FGDs participants mentioned traditional practices done in the area by the mothers especially during the first pregnancy and childbirth. They are assuming that it benefits the mother's health. The community worries about the first birth. One FGD participants expressed the issue as follows:

*In our community, every pregnant woman goes to her mother's home for her first child birth. As the day of delivery approaches relatives and neighbors make the pregnant women smoked by sitting on smoky leaves in fire and they spill butter on her whole body and massage to facilitate spontaneous delivery. A woman misses this important cultural ceremony in health facility.* (IDI, Lemo)

Another FGD participant said that mothers prefer to give birth at home because the placenta has to be buried around their home to avoid evil events and bad luck. Mothers mentioned the issue as follows:

*The placenta should be buried around our living compound, not to be disposed anywhere. But, when mothers give birth in health institution, the health workers will not give us the placenta. Therefore, most of us do not prefer to go to health institution.* (FGD, Lemo)

## 4. Discussion

Qualitative study of this type is crucial to elucidate the experience of mothers giving birth at health facilities and the process involved in the preference of home delivery. This study involved women who gave birth at home to their most recent child despite history of facility delivery.

The main findings were abusive and disrespectful treatment, unskilled care, poor client provider interaction, lack of privacy, and traditional practices. It is disturbing to note that some participants experienced neglectful and arrogant treatment at the health facilities which indeed shaped the mothers' behavior. Several studies have found out the same kind of treatment though there is no universal agreement on how such incidents can be measured and how they occur [16–19]. The reason for such maltreatment could be explained by the fact that professionals in the nation have low degree of job satisfaction; such maltreatments have become routine and/or attitudinal [19, 20]. It has been proved that such treatments are deterrent of facility delivery as it influences women's decision on how, where, and with whom to give childbirth [19, 21]. Abusive and disrespectful treatment violates the enjoyment of the inalienable human rights and ignores the September, 2014, WHO statement let alone its implications for their health and well-being [22]. Moreover, in the study areas, mothers gave birth to six children on average; hence, such treatment during delivery care could lower the government's effort to increase facility based delivery which in turn graves the hope of reducing maternal morbidity and mortality in the region particularly and in the country at large.

This study pointed out that the study participants had poor client provider interaction during childbirth at a health facility. The clients did not receive medically appropriate treatment or care acceptable to them. This finding is consistent with findings in South Africa, Tanzania, and Jordan [15, 23, 24]. The reason for such consistency could be explained by the fact that those countries and Ethiopia have shortage of staffs and poor infrastructure and also professionals have inadequate training and follow-up. Nevertheless, effective communication between the providers and clients is important for the betterment of patient satisfaction and health outcomes. In addition, clients feel confident in the service being provided [25]

Women reported that there was no continuity of care despite the fact that they were hopeful to have continuous care throughout the childbirth. This experience is found to be similar with the study done in Addis Ababa and other developing countries [16, 19, 21]; the reason for intermittent care could be the fact that professionals may monitor more than one labouring woman simultaneously, shift ending or beginning time, being tiresome, and lack of skill on care of women on labour. Nevertheless, studies have proved that continuous care during childbirth is a form of pain relief and empathy and reduces risks of fetal injury [26].

## 5. Conclusion

Health provider's disrespectful treatment and unskilled care are deterring women from seeking skilled attendance at birth. Our findings suggest a need for health care providers to improve client provider relationships and the government also needs to work on health care providers' attitude.

## Figures and Tables

**Table 1:** Categories of themes and subthemes.

Number	Theme	Subthemes
(1)	Facility and provider factors	Abusive and disrespectful treatment
		Unskilled care
		Poor client provider interaction
		Noncontinues care

(2)	Personal factor	Lack of privacy

(3)	Cultural factors	Traditional practices

**Table 2:** Sociodemographic characteristics of women participants, Hadiya zone, Ethiopia, 2016.

Characteristics		Number	Percentages
Age	20–29	61	69.2
	≥30	27	30.8

Education	No education	16	17.6
	Primary	50	57.7
	Secondary	14	15.3
	Above secondary	8	9.4

Number of children	2	11	11.6
	3	30	34.6
	≥4	47	53.8
